# Risk of death due to COVID-19 among current and former smokers in the Netherlands: a population-based quasi-cohort study

**DOI:** 10.1093/ije/dyae003

**Published:** 2024-02-01

**Authors:** Naomi A van Westen-Lagerweij, Marjanne H D Plasmans, Iris Kramer, Peter P M Harteloh, Marinus J J C Poos, Henk B M Hilderink, Esther A Croes

**Affiliations:** The Netherlands Expertise Centre for Tobacco Control, Trimbos Institute, Utrecht, The Netherlands; National Institute for Public Health and the Environment (RIVM), Bilthoven,The Netherlands; The Netherlands Expertise Centre for Tobacco Control, Trimbos Institute, Utrecht, The Netherlands; Department of Health and Care, Statistics Netherlands, The Hague, The Netherlands; National Institute for Public Health and the Environment (RIVM), Bilthoven,The Netherlands; National Institute for Public Health and the Environment (RIVM), Bilthoven,The Netherlands; The Netherlands Expertise Centre for Tobacco Control, Trimbos Institute, Utrecht, The Netherlands

**Keywords:** COVID-19, death, smoking status, population research, the Netherlands

## Abstract

**Background:**

Research on smoking as a risk factor for death due to COVID-19 remains inconclusive, with different studies demonstrating either an increased or decreased risk of COVID-19 death among smokers. To investigate this controversy, this study uses data from the Netherlands to assess the relationship between smoking and death due to COVID-19.

**Methods:**

In this population-based quasi-cohort study, we linked pseudonymized individual data on smoking status from the 2016 and 2020 ‘Health Monitor Adults and Elderly’ in the Netherlands (*n *=* *914 494) to data from the cause-of-death registry (*n *=* *2962). Death due to COVID-19 in 2020 or 2021 was taken as the main outcome. Poisson regression modelling was used to calculate relative risks (RRs) and 95% CIs of death due to COVID-19 for current and former smokers compared with never smokers while adjusting for relevant confounders (age, sex, educational level, body mass index and perceived health).

**Results:**

Former smokers had a higher risk of death due to COVID-19 compared with never smokers across unadjusted (RR, 2.22; 95% CI, 2.04–2.42), age–sex-adjusted (RR, 1.38; 95% CI, 1.22–1.55) and fully adjusted (RR, 1.30; 95% CI, 1.16–1.45) models. Current smokers had a slightly higher risk of death due to COVID-19 compared with never smokers after adjusting for age and sex (RR, 1.21; 95% CI, 1.00–1.48) and after full adjustment (RR, 1.08; 95% CI, 0.90–1.29), although the results were statistically non-significant.

**Conclusions:**

People with a history of smoking appear to have a higher risk of death due to COVID-19. Further research is needed to investigate which underlying mechanisms may explain this.

Key Messages

- After adjusting for relevant confounders, we found that, compared with never smokers, former smokers had a moderately increased risk of death due to COVID-19 (30%).
- Among current smokers, there was a statistically non-significant trend towards a small increased risk of death due to COVID-19 (8%), indicating that the risk of COVID-19 death may not significantly differ between current smokers and never smokers.
- The findings of this study do not point to a protective effect of smoking on COVID-19 death, as suggested by several previous studies.
- Further research is needed to investigate which mechanisms may explain a higher risk of COVID-19 death for former smokers and no increased risk of COVID-19 death for current smokers compared with never smokers.

## Introduction

As of January 2023, the coronavirus disease 2019 (COVID-19) had caused >6.6 million deaths worldwide.^1^ Several risk factors of COVID-19-related mortality (i.e. death among people with COVID-19) have been identified in the literature, including older age, male sex and the presence of chronic diseases.^2–4^ Although smoking has also been linked to COVID-19-related mortality,^2^ the literature with regard to this risk factor remains inconclusive.

Three large meta-analyses found that former smokers and people with a history of smoking (i.e. former and current smokers combined) had an increased risk of COVID-19-related mortality compared with never smokers^5–7^ but no statistically significant association was found for current smokers.^5^^,^^6^ Important to note is that two of these three meta-analyses mainly included studies that did not adjust for confounders such as age and sex.^5^^,^^6^ In addition, the three meta-analyses mostly included hospitalized patients, which is problematic as several biases potentially exist in hospital data. For example, hospitalization may result in the abrupt quitting of smoking and thus the misclassification of current smokers as former smokers. Also, as both smoking and COVID-19 death may be associated with hospitalization, the association between smoking and COVID-19 death may be distorted among hospitalized COVID-19 patients.

Using national death registrations linked to data on smoking status (collected outside the hospital setting) is less prone to the above-mentioned biases. Only a few studies have used such data to determine the association between smoking status and COVID-19-related mortality while adjusting for relevant confounders. However, the results are ambiguous. Two studies from the UK, which both used data from the same UK Biobank cohort, found that both current and former smoking were associated with a higher risk of COVID-19-related death^8^^,^^9^ and that this association was strongest among older current smokers^8^ and heavy current smokers.^9^ Important to note is that the information on smoking status was collected during the UK Biobank recruitment period between 2006 and 2010, meaning that the information may be (partly) outdated. Two other studies, which both used more recent data on smoking status from the OpenSAFELY platform in the UK (based on electronic patient records from general practices), found that, although current and former smoking was associated with a higher risk of COVID-19-related death, current smokers had a lower risk of COVID-19-related death than former smokers.^4^^,^^10^ Also, the association of current smoking with non-COVID-19-related death was greater than the association with COVID-19-related death.^10^ Finally, a fifth study from the UK that also used patient data from general practices found that current smoking, compared with never smoking, was associated with a one-fifth lower risk of death due to COVID-19.^11^

In this study, we use data on death due to COVID-19 from the Dutch national cause-of-death registry and data on smoking collected through a population-based health survey to assess the relationship between smoking and death due to COVID-19 in the Netherlands.

## Methods

### Study design

In this population-based quasi-cohort study, we linked pseudonymized individual data from the 2016 and 2020 ‘Health Monitor Adults and Elderly’ to pseudonymized individual data from the Dutch cause-of-death registry, resulting in what we describe as a quasi-cohort. The Health Monitor is a national questionnaire of the Dutch Municipal Public Health Service (GGD), Statistics Netherlands (CBS) and the National Institute for Public Health and the Environment (RIVM). It is used to provide an overview of the health, wellbeing and lifestyle of (non-institutionalized) residents in the Netherlands aged ≥18 years. Every 4 years, a representative sample of >1 million people is selected by CBS from the Personal Records Database (BRP) and asked to fill out the Health Monitor, either online or on paper. The BRP includes personal data of all residents in the Netherlands. In 2016 and 2020, ∼540 000 and ∼460 000 respondents, respectively, completed the Health Monitor.

The cause-of-death registry contains all causes of death of deceased persons in the Netherlands. For each deceased person in the Netherlands, a cause-of-death certificate is completed by a physician. The certificates are processed by CBS. For each deceased person, one underlying cause of death is selected and all other causes reported are coded as contributing to death. World Health Organization (WHO) guidelines are used for coding and selecting the underlying cause of death. For COVID-19, the WHO issued an instruction that COVID-19 should be assigned as underlying cause of death if COVID-19 is included in the first part of the death certificate, unless it involves an accident, suicide or violence (i.e. external cause of death).^12^

**Figure 1.**
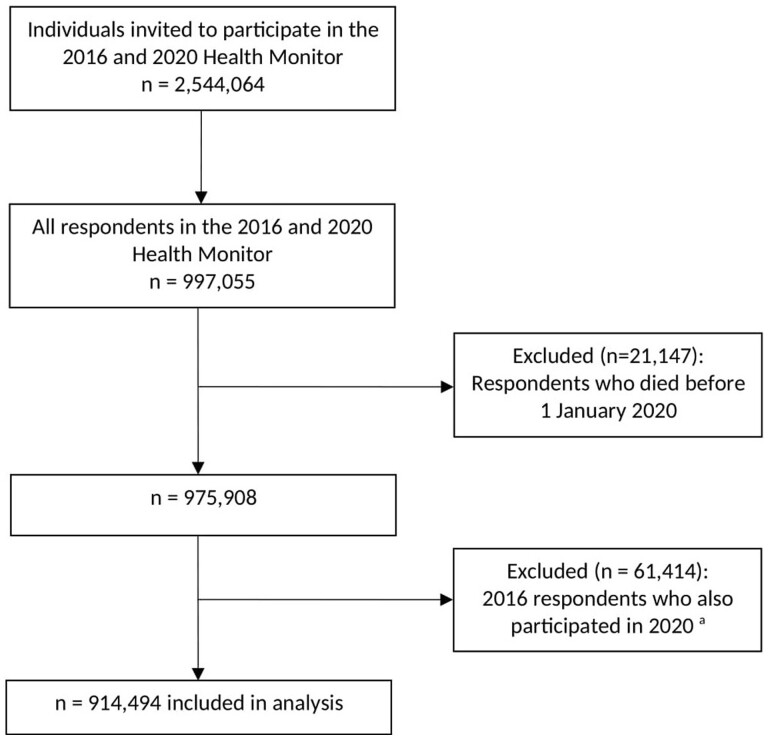
Participant flowchart. ^a^Among the 61 414 excluded respondents who participated in both years, about two-thirds were aged >65 years. Also, 67.5% of these excluded respondents who were current smokers in 2016 were still current smokers in 2020, and 89.7% of the excluded respondents who were former smokers in 2016 were still former smokers in 2020

For this study, we included respondents who completed the Health Monitor in 2016 or 2020 and who were alive on 1 January 2020. We excluded respondents from the 2016 Health Monitor who had also completed the Health Monitor in 2020. For these respondents, we only used the information from 2020. See Figure 1 for the participant flowchart.

### Measures

#### Outcome

The main outcome variable used in the analysis was death due to COVID-19—that is, any death in 2020 or 2021 in the cause-of-death registry with the International Classification of Diseases 10th Revision (ICD-10) underlying cause code of U07.1 (‘COVID-19, virus identified’) or U07.2 (‘COVID-19, virus not identified’).^12^

#### Independent variable

We used the data from the Health Monitor on smoking status. Smoking status was classified as current smoker, former smoker or never smoker. Current smokers were defined as those who answered ‘yes’ to the question: ‘Do you sometimes smoke?’ In the questionnaire, it was explained that this included smoking all types of tobacco products, except for electronic cigarettes and devices in which tobacco is heated (heat-not-burn). Former smokers were defined as those who answered ‘yes’ to the question: ‘Did you smoke in the past?’ Respondents who answered ‘no’ to both questions were considered to be never smokers.

#### Covariates

We included several covariates that may be associated with both smoking status and COVID-19-related mortality, using data from the Health Monitor.

*Demographics.* Demographic variables included age, sex and educational level. For ‘educational level’, we used the highest level of education attained. We categorized ‘educational level’ into low (no education, elementary school), lower middle (lower secondary education, lower vocational education), upper middle (intermediate vocational education, higher secondary education) and high (higher vocational education or university).

*Overweight.* The body mass index (BMI) of each respondent was determined based on self-reported height and weight. BMI was categorized into ‘no overweight’ (<25 kg/m^2^), ‘moderate overweight’ (25–30 kg/m^2^) and ‘severe overweight’ (>30 kg/m^2^).

*Perceived health.* Respondents were asked to answer the question ‘How is your health in general?’ using a five-point Likert scale. We created three categories: ‘very good or good’, ‘not bad’ and ‘poor or very poor’.

### Statistical analyses

Statistical analyses were performed using R Statistical Software (version 3.6.2). We used Poisson regression modelling to calculate relative risks (RRs) and 95% CIs of death due to COVID-19 for smokers and former smokers compared with never smokers. We ran three models: an unadjusted model, an age–sex-adjusted model and a fully adjusted model that included age, sex, educational level, overweight and perceived health. Because the residual deviance was greater than the degrees of freedom, we adjusted the model for overdispersion by using quasi-Poisson regression models. All included variables were nominal variables with a reference group. We additionally included an offset variable with the log of the population size. Individuals with missing data were excluded from the analyses (complete case analysis). A total of 48 735 individuals with missing data were excluded from the unadjusted model; in the age-adjusted model, 55 783 individuals with missing data were excluded; and in the fully adjusted model, 124 865 individuals with missing data were excluded.

We additionally conducted two sensitivity analyses. The first sensitivity analysis included a fully adjusted regression model with death due to lung cancer (ICD-10 codes C33 and C34) as the outcome. The purpose of this analysis was to verify the reliability of the data on smoking status, which is known to be strongly related to dying from lung cancer. In the second sensitivity analysis, we ran four fully adjusted regression models with death due to COVID-19 as the outcome in which we distinguished between four different periods during the first 2 years of the COVID-19 pandemic. We hypothesized that the RR of death due to COVID-19 may have changed during the COVID-19 pandemic as a result of immunity and different variants of the severe acute respiratory syndrome coronavirus 2 (SARS-CoV-2 virus). From the beginning of 2020 until the end of July 2020 (i.e. Period 1), the original SARS-CoV-2 virus spread in the Netherlands and only few had developed natural immunity against the virus. During this period, testing capacity was limited and the diagnosis was mostly based on a clinical suspicion of COVID-19 (ICD-10 code U07.2 in the cause-of-death registry). Between August 2020 and the end of November 2020 (i.e. Period 2), the original virus was still circulating and natural immunity was slowly growing among the Dutch population. Testing capacity increased and the number of deaths with a U07.2 code decreased. Between December 2020 and the end of June 2021 (i.e. Period 3), the Alpha variant of the virus was dominant and the Dutch population started to get vaccinated. Between July 2021 and the end of December 2021 (i.e. Period 4) the Delta variant of the virus was dominant and the majority of the Dutch population was vaccinated.

## Results

A total of 914 494 respondents were included in the analyses (Figure 1). Among these respondents, 43.2% were never smokers, 42.0% were former smokers and 14.8% were current smokers (Table 1). Most respondents were female (53.8%), aged <70 years (59.1%) and had attained high or upper middle education (64.0%). The characteristics of the respondents stratified by smoking status are presented in Supplementary Table S1 (available as Supplementary data at *IJE* online).

**Table 1.** Cohort characteristics

Variable and category	Total population [*n* (%)]	COVID-19 deaths [*n* (%)]
Smoking status		
Never smoker	374 262 (43.2)	785 (29.0)
Former smoker	363 381 (42.0)	1696 (62.6)
Current smoker	128 116 (14.8)	229 (8.5)
Unknown	48 735	252
Sex		
Female	491 810 (53.8)	1139 (38.5)
Male	422 684 (46.2)	1823 (61.5)
Age (years)		
18–64	433 128 (48.1)	73 (2.5)
65–69	98 668 (11.0)	103 (3.5)
70–74	132 680 (14.7)	309 (10.5)
75–79	105 863(11.8)	517 (17.6)
80–84	70 530 (7.8)	656 (22.3)
85–89	39 854 (4.4)	683 (23.3)
90–94	15 938 (1.8)	416 (14.2)
95+	3991 (0.4)	179 (6.1)
Unknown	13 842	26
Educational level		
High	276 919 (32.3)	355 (13.3)
Upper middle	271 591 (31.7)	550 (20.5)
Lower middle	257 524 (30.1)	1208 (45.1)
Low	50 161 (5.9)	564 (21.1)
Unknown	58 299	285
Overweight		
No overweight	415 337 (47.1)	933 (34.8)
Moderate overweight	332 980 (37.7)	1055 (39.3)
Severe overweight	134 015 (15.2)	696 (25.9)
Unknown	32 162	278
Perceived health		
Very good or good	683 373 (75.4)	1068 (36.5)
Not bad	191 398 (21.1)	1400 (47.8)
Poor or very poor	31 565 (3.5)	459 (15.7)
Unknown	8158	35

A total of 2962 respondents died of COVID-19 in 2020 or 2021 (Table 1). Compared with the total population of respondents, those who had died of COVID-19 were more often former smokers (62.6%), male (61.5%), ≥70 years old (94.0%) and had attained lower middle or low education (66.2%) (Table 1).

Table 2 shows the unadjusted and adjusted RRs and 95% CIs of death due to COVID-19 for the independent variable (smoking status) and each covariate. Former smokers had a greater risk of death due to COVID-19 compared with never smokers across unadjusted (RR, 2.22; 95% CI, 2.04–2.42), age–sex-adjusted (RR, 1.38; 95% CI, 1.22–1.55) and fully adjusted (RR, 1.30; 95% CI, 1.16–1.45) models. Current smokers had a lower risk of death due to COVID-19 compared with never smokers in the unadjusted model (RR, 0.85; 95% CI, 0.74–0.99). After adjusting for age and sex, current smokers had a statistically non-significant higher risk of death due to COVID-19 compared with never smokers (RR, 1.21; 95% CI, 1.00–1.48). In the fully adjusted model, current smokers still had a slightly higher risk of death due to COVID-19 compared with never smokers (RR, 1.08; 95% CI, 0.90–1.29), although the results remained statistically non-significant.

**Table 2.** Unadjusted and adjusted relative risks of death due to COVID-19

	Model 1: unadjusted (*n*=865 759)		Model 2: age–sex-adjusted (*n*=858 711)		Model 3: fully adjusted (*n*=789 629)	
	RR (95% CI)	*P*	RR (95% CI)	*P*	RR (95% CI)	*P*
**Independent variable**						
Smoking status						
Never smoker	Ref.		Ref.		Ref.	
Former smoker	2.22 (2.04–2.42)	<0.001	1.38 (1.22–1.55)	<0.001	1.30 (1.16–1.45)	<0.001
Current smoker	0.85 (0.74–0.99)	0.033	1.21 (1.00–1.48)	0.060	1.08 (0.90–1.29)	0.431
**Covariates**						
Sex						
Female			Ref.		Ref.	
Male			1.73 (1.56–1.93)	<0.001	2.11 (1.90–2.34)	<0.001
Age, years						
18–64			Ref.		Ref.	
65–69			5.60 (3.74–8.40)	<0.001	5.05 (3.47–7.34)	<0.001
70–74			12.84 (9.11–18.11)	<0.001	10.93 (7.91–15.10)	<0.001
75–79			26.01 (18.67–36.24)	<0.001	20.59 (15.05–28.18)	<0.001
80–84			50.77 (36.60–70.45)	<0.001	38.37 (28.12–52.37)	<0.001
85–89			96.99 (69.94–134.48)	<0.001	67.75 (49.59–92.56)	<0.001
90–94			154.52 (110.29–216.49)	<0.001	105.54 (76.40–145.79)	<0.001
95+			281.24 (194.09–407.51)	<0.001	185.23 (129.30–265.36)	<0.001
Educational level						
High					Ref.	
Upper middle					1.41 (1.21–1.66)	<0.001
Lower middle					1.69 (1.47–1.96)	<0.001
Low					2.06 (1.74–2.44)	<0.001
Overweight						
No overweight					Ref.	
Moderate overweight					1.07 (0.96–1.19)	0.223
Severe overweight					1.73 (1.53–1.96)	<0.001
Perceived health						
Very good or good					Ref.	
Not bad					2.28 (2.05–2.53)	<0.001
Poor or very poor					4.48 (3.88–5.17)	<0.001

RR, relative risk.

The adjusted models showed that age is the strongest predictor of death due to COVID-19, with the RR of death due to COVID-19 increasing with age [e.g. for age 95+ years, compared with 18–64 years, the RR was 185.23 (95% CI, 129.30–265.36)]. Other risk factors of death due to COVID-19 were male sex (RR, 2.11; 95% CI, 1.90–2.34), upper middle education (vs high education: RR, 1.41; 95% CI, 1.21–1.66), lower middle education (vs high education: RR, 1.69; 95% CI, 1.47–1.96), low education (vs high education: RR, 2.06; 95% CI, 1.74–2.44), severe overweight (RR, 1.73; 95% CI, 1.53–1.96), ‘not bad’ perceived health (vs ‘very good or good’ perceived health: RR, 2.28; 95% CI, 2.05–2.53) and ‘poor or very poor’ perceived health (vs ‘very good or good’ perceived health: RR, 4.48; 95% CI, 3.88–5.17).

The sensitivity analyses show that both former smokers (RR, 6.73; 95% CI, 5.23–8.67) and current smokers (RR, 16.77; 95% CI, 12.92–21.76) had a greater risk of death due to lung cancer compared with never smokers (Supplementary Table S2, available as Supplementary data at *IJE* online).

Also, former smokers had a greater risk of death due to COVID-19 during Period 1 (RR, 1.43; 95% CI, 1.14–1.78) and Period 3 (RR, 1.30; 95% CI, 1.11–1.53) of the pandemic compared with never smokers (Supplementary Table S2, available as Supplementary data at *IJE* online). We found no statistically significant results for former smokers in Period 2 (RR, 1.27; 95% CI, 0.95–1.68) and Period 4 (RR, 1.21; 95% CI, 0.97–1.51) of the pandemic. The results for current smokers remained statistically non-significant across the four periods, with the highest RR observed during Period 1 of the pandemic (RR, 1.27; 95% CI, 0.88–1.83).

## Discussion

After adjusting for relevant confounders, we found that, compared with never smokers, current smokers had a statistically non-significant trend towards a small increased risk of death due to COVID-19 (8%) and former smokers had a moderately increased risk of death due to COVID-19 (30%). Although this finding may imply that the risk of death due to COVID-19 does not differ between current smokers and never smokers, there may also be two other explanations: residual confounding may distort the observed association and/or the number of smokers among the people who died from COVID-19 was too small to demonstrate a statistically significant difference. As age is the strongest predictor of death due to COVID-19 and the prevalence of current smoking decreases with age (as shown in Supplementary Table S1, available as Supplementary data at *IJE* online), a substantially larger data set (i.e. at least seven times larger, under the assumption that the RR remains unchanged) would be required to demonstrate a possible statistically significant association between current smoking and death due to COVID-19.

Our results are consistent with the findings of two other studies in the UK which found that current smokers had a lower risk of COVID-19-related death than former smokers.^4^^,^^10^ In line with the results of other studies, we also found that older age, male sex and severe overweight were associated with an increased risk of death due to COVID-19.^2–4^ Although several biological explanations for these different risk factors have been identified in the literature,^13–16^ there is currently no strong evidence for biological mechanisms that may explain a lower risk of death for current smokers vs former smokers (such as a protective effect of nicotine).^17^ In fact, there is strong evidence that smoking is associated with lung damage, impaired immune response, endothelial dysfunction and cardiovascular disease, which in turn may negatively affect the course of COVID-19, including leading to death.^17^ A large meta-analysis, however, concluded that the incidence of SARS-CoV-2 infection is generally lower among current smokers compared with never smokers.^6^ If also true in our study population, this means that a low infection rate may have weakened the risk of COVID-19 death for current smokers, thus explaining the lower risk of COVID-19 death for current smokers. Further research is, however, necessary to determine whether or not smokers have a reduced risk of SARS-CoV-2 infection.^17^

Although we found few statistically significant associations when we ran the analyses for the four different periods of the COVID-19 pandemic, we did find that most point estimates pointed in the direction of an increased risk of death due to COVID-19 among current and former smokers. The highest increased risk of death due to COVID-19 was observed during the first period of the pandemic (27% for current smokers, though statistically non-significant, and 43% for former smokers). More research with data from other countries is needed to confirm whether the SARS-CoV-2 virus was indeed more deadly for current and former smokers at the beginning of the pandemic.

### Strengths and limitations

This was the first study to use data from the Netherlands to assess the relationship between smoking status and death due to COVID-19 and, as such, allows comparisons between countries. A strength of this study is that the data were collected from a large sample of the Dutch population. Also, smoking status was directly reported by respondents themselves and not, for example, extracted from electronic patient records, which are often incomplete or contain errors with regard to smoking status.^18–20^ Several limitations of this study should, however, be acknowledged. First, although we adjusted for important confounders such as age, sex and educational level, we were unable to adjust for other variables that may also influence the relationship between smoking status and death due to COVID-19, such as the presence of chronic illnesses caused by smoking, vaccination status and SARS-CoV-2 variant. We did, however, adjust for ‘perceived health’, which can be seen as a proxy for underlying health issues, and in our sensitivity analyses we partly accounted for vaccination status and variants of the virus by distinguishing between different periods of the pandemic. More research is, however, needed to investigate how vaccination status and virus variant may have influenced the risk of death due to COVID-19 among current and former smokers. Second, we were limited in our definition of smoking status. The Health Monitor only included the question ‘Do you sometimes smoke?’ to identify current smokers and ‘Did you smoke in the past?’ to identify former smokers. It is possible that individuals who have smoked once in their lifetime answered ‘yes’ to one of these questions. We unfortunately lacked data on how many cigarettes someone had smoked in the past 30 days (for current smokers) or past (for former smokers). Such data would result in a more precise definition of current and former smokers, and would allow us to distinguish between light and heavy smokers. Third, as smoking status was recorded in 2016 and 2020, it is possible that smokers quit smoking in the meantime. The influence of misclassification is, however, expected to be small. Our sensitivity analysis showed a strong relationship between smoking status and death due to lung cancer, indicating that the data with regard to smoking status are reliable and likely not strongly biased by misclassification. Fourth, the Health Monitor only includes non-institutionalized residents in the Netherlands, meaning that people living in nursing or care homes, mental health institutions and institutions for the mentally disabled were not included in the study population. Lastly, the validity of the dependent variable (i.e. death due to COVID-19) may have been influenced by the special WHO instruction to code COVID-19 as the underlying cause of death when COVID-19 is mentioned in the first part of the death certificate. This instruction was motivated by surveillance and not by causal considerations. Application of the general principle for cause-of-death statistics (i.e. the start of the causal chain of morbid events is the underlying cause of death) might change the distribution of smoking among deaths due to COVID-19 in the Netherlands.

## Conclusions

The results of this study show that people with a history of smoking had a higher risk of death due to COVID-19 in 2020 and 2021 than never smokers. Current smokers, however, did not have an increased risk of death due to COVID-19 compared with never smokers. Further research is needed to confirm this unexpected finding and to investigate which mechanisms may explain such a finding.

## Ethics approval

Ethics approval was not required, as this study relied on secondary pseudonymized data. According to Dutch Civil Law (Article 7:458), no ethics approval is required for a secondary analysis of non-identifiable data. Results reported in this paper cannot be traced back to individual persons. The study was conducted in line with the Declaration of Helsinki and applicable laws on privacy.

## Supplementary Material

dyae003_Supplementary_Data

## Data Availability

The data underlying this article cannot be shared publicly due to Dutch privacy laws.
