# Supplementary material for: Risk of death due to COVID-19 among current and former smokers in the Netherlands: a population-based quasi-cohort study
Source: Int J Epidemiol. 2024 Feb 1;53(1):dyae003. doi: 10.1093/ije/dyae003 (PMC10834359; doi:10.1093/ije/dyae003)
Supplement: dyae003_Supplementary_Data [file dyae003_supplementary_data.docx]

| **Variable** | **Category** | **Never smoker**  **No. (%)** | **Former smoker**  **No. (%)** | **Current smoker**  **No. (%)** | **Unknown**  **No.** |
| --- | --- | --- | --- | --- | --- |
| Gender | Female | 227,711 (49.3) | 173,349 (37.5) | 61,196 (13.2) | 29,554 |
|  | Male | 146,551 (36.3) | 190,032 (47.1) | 66,920 (16.6) | 19,181 |
| Age | 18-64 | 219,434 (52.4) | 121,120 (28.9) | 78,245 (18.7) | 14,329 |
|  | 65-69 | 28,946 (30.5) | 51,746 (54.5) | 14,215 (15.0) | 3,761 |
|  | 70-74 | 39,613 (31.4) | 70,311 (55.8) | 16,046 (12.7) | 6,710 |
|  | 75-79 | 34,255 (34.4) | 55,353 (55.6) | 9,937 (10.0) | 6,318 |
|  | 80-84 | 25,466 (39.1) | 34,252 (52.6) | 5,415 (8.3) | 5,397 |
|  | 85-89 | 15,067 (41.4) | 19,006 (52.2) | 2,325 (6.4) | 3,456 |
|  | 90-94 | 6,386 (44.3) | 7,409 (51.4) | 620 (4.3) | 1,523 |
|  | 95+ | 1,902 (53.7) | 1,536 (43.3) | 106 (3.0) | 447 |
|  | Unknown | 3,193 | 2,648 | 1,207 | 6,794 |
| Educational level | High | 136,258 (50.8) | 102,884 (38.3) | 29,250 (10.9) | 8,527 |
|  | Upper middle | 111,354 (42.6) | 105,518 (40.4) | 44,227 (16.9) | 10,492 |
|  | Lower middle | 88,254 (36.4) | 116,746 (48.1) | 37,761 (15.6) | 14,763 |
|  | Low | 18,310 (39.7) | 19,485 (42.3) | 8,323 (18.0) | 4,043 |
|  | Unkown | 20,086 | 18,748 | 8,555 | 10,910 |
| Overweight | No overweight | 190,984 (48.0) | 142,635 (35.9) | 64,218 (16.1) | 17,500 |
|  | Moderate overweight | 124,598 (39.2) | 149,491 (47.0) | 44,126 (13.9) | 14,765 |
|  | Severe overweight | 48,666 (38.1) | 62,284 (48.8) | 16,812 (13.2) | 6,253 |
|  | Unknown | 10,014 | 8,971 | 2,960 | 10,217 |
| Perceived health | Very good or good | 296,624 (45.7) | 262,964 (40.5) | 90,138 (13.9) | 33,647 |
|  | Not bad | 65,272 (36.3) | 83,874 (46.7) | 30,474 (17.0) | 11,778 |
|  | Poor or very poor | 9,596 (32.6) | 13,464 (45.8) | 6,364 (21.6) | 2,141 |
|  | Unknown | 2,770 | 3,079 | 1,140 | 1,169 |

**Supplementary Table S1.** Characteristics of total population (n=914,494) stratified by smoking status.

**Supplementary Table S2.** Results from the sensitivity analyses: Fully adjusted relative risk of lung cancer-related mortality, and fully adjusted relative risks of COVID-19-related mortality stratified by period.

|  |  | **Lung cancer-related mortality** | **COVID-19-related mortality – Period 1**  **(01.01.20-31.06.20)** | **COVID-19-related mortality – Period 2**  **(01.08.20-30.11.20)** | **COVID-19-related mortality – Period 3**  **(01.12.20-30.06.21)** | **COVID-19-related mortality – Period 4 (01.07.21-31.12.21)** |
| --- | --- | --- | --- | --- | --- | --- |
| *Independent variable* | | RR [95%CI] | RR [95%CI] | RR [95%CI] | RR [95%CI] | RR [95%CI] |
| Smoking status | Never smoker | ref | ref | ref | ref | ref |
|  | Former smoker | **6.73 [5.23-8.67]** | **1.43 [1.14-1.78]** | 1.27 [0.95-1.68] | **1.30 [1.11-1.53]** | 1.21 [0.97-1.51] |
|  | Current smoker | **16.77 [12.92-21.76]** | 1.27 [0.88-1.83] | 1.07 [0.67-1.72] | 0.92 [0.69-1.22] | 1.24 [0.88-1.76] |
| *Covariates* |  |  |  |  |  |  |
| Gender | Female | ref | ref | ref | ref | ref |
|  | Male | **1.34 [1.19-1.51]** | **2.13 [1.74-2.62]** | **2.65 [2.02-3.47]** | **2.05 [1.76-2.38]** | **1.99 [1.62-2.44]** |
| Age | 18-64 | ref | ref | ref | **ref** | ref |
|  | 65-69 | **3.74 [2.91-4.81]** | **4.67 [2.00-10.89]** | **3.75 [1.11-12.72]** | **4.71 [2.91-7.61]** | **7.41 [3.14-17.45]** |
|  | 70-74 | **5.70 [4.55-7.13]** | **12.18 [5.96-24.87]** | **14.12 [5.33-37.39]** | **8.67 [5.70-13.19]** | **16.42 [7.64-35.29]** |
|  | 75-79 | **7.59 [6.06-9.51]** | **24.44 [12.23-48.85]** | **29.61 [11.49-76.29]** | **15.60 [10.39-23.41]** | **30.77 [14.55-65.04]** |
|  | 80-84 | **9.51 [7.52-12.04]** | **47.98 [24.16-95.26]** | **62.66 [24.55-159.95]** | **25.75 [17.18-38.61]** | **64.22 [30.61-134.75]** |
|  | 85-89 | **12.04 [9.32-15.56]** | **103.52 [52.29-204.94]** | **109.18 [42.66-279.43]** | **43.38 [28.84-65.25]** | **105.46 [50.08-222.10]** |
|  | 90-94 | **11.73 [8.36-16.47]** | **146.24 [72.31-295.73]** | **130.51 [49.21-346.15]** | **74.81 [48.93-114.38]** | **176.76 [82.62-378.15]** |
|  | 95+ | **4.97 [1.93-12.78]** | **281.20 [131.56-601.07]** | **220.21 [75.27-644.18]** | **154.67 [96.37-248.21]** | **209.48 [88.61-495.19]** |
| Educational level | High | ref | ref | ref | ref | ref |
|  | Upper middle | **1.46 [1.21-1.76]** | 1.26 [0.93-1.71] | 1.27 [0.84-1.90] | **1.57 [1.24-1.99]** | 1.38 [1.00-1.90] |
|  | Lower middle | **1.70 [1.44-2.02]** | **1.38 [1.04-1.82]** | **1.58 [1.10-2.28]** | **1.91 [1.54-2.38]** | **1.70** **[1.27-2.28]** |
|  | Low | **1.75 [1.40-2.19]** | **1.84 [1.34-2.53]** | **2.34 [1.56-3.53]** | **2.09** [**1.62-2.71]** | **2.07 [1.48-2.90]** |
| Overweight | No overweight | ref | ref | ref | ref | ref |
|  | Moderate overweight | **0.79 [0.70-0.90]** | 1.16 [0.94-1.44] | 1.29 [0.98-1.71] | 1.02 [0.87-1.20] | 0.98 [0.79-1.22] |
|  | Severe overweight | **0.71 [0.60-0.85]** | **1.81 [1.42-2.32]** | **1.99 [1.45-2.73]** | **1.76 [1.47-2.10]** | **1.51 [1.18-1.93]** |
| Experienced health | Very good or good | ref | ref | ref | ref | ref |
|  | Okay | **1.99 [1.74-2.27]** | **2.43 [1.98-2.97]** | **2.44 [1.88-3.17]** | **2.14 [1.83-2.49]** | **2.37 [1.92-2.92]** |
|  | Bad or very bad | **5.90 [5.02-6.95]** | **3.51 [2.58-4.79]** | **4.34 [2.99-6.31]** | **4.75 [3.86-5.85]** | **5.14 [3.87-6.81]** |

Note: Bold values denote statistical significance at the p < 0.05 level.
